# Microfluidic implementation of functional cytometric microbeads for improved multiplexed cytokine quantification

**DOI:** 10.1063/1.5044449

**Published:** 2018-08-10

**Authors:** Ya Liu, Jiyu Li, Dinglong Hu, Josh H. M. Lam, Dong Sun, Stella W. Pang, Raymond H. W. Lam

**Affiliations:** 1 Department of Mechanical and Biomedical Engineering, City University of Hong Kong, Hong Kong 999077, China; 2 Centre for Robotics and Automation, City University of Hong Kong, Hong Kong 999077, China; 3 Centre for Biosystems, Neuroscience, and Nanotechnology, City University of Hong Kong, Hong Kong 999077, China; 4 Department of Electronic Engineering, City University of Hong Kong, Hong Kong 999077, China; 5 City University of Hong Kong Shenzhen Research Institute, Shenzhen 518057, China

## Abstract

Functional microbeads have been widely applied in molecular identification and other biochemical applications in the past decade, owing to the compatibility with flow cytometry and the commercially available microbeads for a wide range of molecular identification. Nevertheless, there is still a technical hurdle caused by the significant sample volume required (∼50 *μ*l), limited molecular detection limit (∼20 pg/ml), complicated liquid/microbead handling procedures, and the long reaction time (>2 h). In this work, we optimize the operation of an automated microbead-based microfluidic device for the reagent mixing and the dynamic cytokine detection. In particular, we adopt fluorescence microscopy for quantification of multiple microbeads in each microchamber instead of flow cytometry for a lower detection limit. The operation parameters are then configured for improved measurement performance. As demonstrated, we consider the cytokine secretion of human macrophage-differentiating lymphocytes stimulated by lipopolysaccharides. We examine requirements on the mixing duration, minimal sample volume, and the image analysis scheme for the smaller biosample volume (<5 *μ*l), the lower cytokine detection limit (∼5 pg/ml), and shorter process time (∼30 min). Importantly, this microfluidic strategy can be further extended in the molecular profiling using other functional microbeads for a broad range of biomedical applications.

## INTRODUCTION

Functional microbeads have been widely used as biochemical probes in biomedical applications, due to the advantages such as relatively high intensity,^1,2^ long-term luminescence stability,^3^ uniformity,^1^ and large sensing surface area.^4–6^ Shibata *et al.* applied fluorescence microbeads for measuring glucose and monitor diabetes status *in vivo.*^7^ Wu *et al.* demonstrated the diagnosis of lung cancer by applying multi-color quantum dots and microbeads.^8^ There are also commercially available functional microbeads, which are coated with specific antibody for conjugating the target antigen in a biosample^9^ for applications such as cell surface protein expression, molecular secretion identification, and bacteria counting.^10^ These functional microbeads are usually designed for the flow cytometry as a highly sensitive detection scheme. For example, Rufer *et al.* developed a flow cytometry scheme of microbeads to test the telomere length for revealing the replicative tendency of lymphocytes^11^ and implicating the possible inflammatory status. Liu *et al.* devised multi-functional microbeads for the simultaneous determination of multiple DNA and protein biomolecules using flow cytometry.^12^

Importantly, the mixture of different kinds of functional microbeads can offer a multiplex detection mode and support the quantification of multiple simultaneously, varying the biochemical characteristics. In particular, the multiplex detection matches perfectly with the necessary requirements of immune system diagnosis, in which multiple cytokine levels from the same sample have to be quantified individually. Although many other conventional gold standard techniques, such as the enzyme-linked immunosorbent assay (ELISA)^13^ and enzyme-linked immunospot assay (ELISPOT),^14–18^ have also been modified and improved as immunoassays, the multiplex microbead detection mode has an outstanding performance in terms of the high throughput and sensitivity.^19^ Hildesheim *et al.* applied the functional microbeads to test hundreds of specimens and proved the reliability of multi-array microbeads in determining the cytokine levels of multiple samples with a low volume.^10^ While very effective, the multiplex microbeads with flow cytometer still need further improvements in terms of the large sample volume, long labeling time, and complicated washing process.^9^

Microfluidics is an ideal strategy for processing the microparticles and liquid biosamples for the microbead-based detection, with the great reduction in reagent consumption and automation of tedious liquid and microbead handling^20,21^ with very high precision and consistency.^22–26^ Further, mixing in microfluidics can effectively speed up the transport rates and reactions of reagents and biomolecules.^27–29^ Han *et al.* reported a quantitative microengraving strategy capable of recording the rates of cytokine secretion from stimulated human peripheral blood mononuclear cells.^30^ In fact, there are already microfluidic techniques integrating with the microbead sensing schemes.^31–34^ Frisk *et al.* presented a microfluidic device embedded with silica beads to monitor the proteolytic activity through signal amplification.^33^ Choi *et al.* developed a microfluidic biochemical testing system based on magnetic beads' protein analysis and bio-molecule detection.^35^ Zhang *et al.* developed a microfluidic chip with quantum dot for the Hepatitis B virus genotyping at a lower detection limit.^36^ An immunoassay platform integrated with the antibody-conjugated microbeads was designed by Han *et al.* for the immunobinding assays and the identification of cancer marker.^37^ Similarly, Zhu *et al.* reported a microparticle array for the detection of human chorionic gonadotropin and prostate specific antigen in serum samples, potentially for the point-of-care diagnosis of diseases.^38^

Many of the reported microbead-based microfluidic devices support the molecular expression at only one time point, rather than profiling their transient dynamics. For example, the dynamic variations of cytokine levels in the body reflect more representatively the corresponding immune status and immune diseases.^39^ Recently, we have reported a microfluidic mixing-assisted multiplexed dynamic cytokine profiling strategy compatible with the commercially available cytometric microbeads,^40^ yet the configuration parameters for the liquid handling, reagent mixing, and measurement performance have not been optimized. This work describes the required procedures and expected results of configuring the measurement parameters (e.g., reagent mixing time and image analysis strategy) for the multiplex molecular quantification with the reduced detection limit and process time. By performing the same procedures, such microbead microfluidic strategy can be further applied for the detection using any other functional microbeads. In addition, we demonstrate the feasibility and performance of the microfluidic microbead-based measurement on the dynamic cytokine secretion from the human monocytic leukemia cells stimulated with lipopolysaccharides.

## MATERIALS AND METHODS

### Cell culture

Frozen human monocytic leukemia cells (THP-1) were thawed by gentle agitation in water bath at 37 °C and were transferred into a centrifuge tube containing 9.0 ml culture medium [90% Roswell Park Memorial Institute (RPMI)-1640, 10% fetal bovine serum (FBS), and 0.05 mM 2-mercaptoethanol]. The tube was spun at 125×g for 5 min. The cells were re-suspended with the medium (volume: 5 ml) and cultured in a 10 cm^2^ flask. All cells were maintained in an incubator at 37 °C supplied with 5% CO_2_.

Before stimulation and cytokine detection, the promonocytic THP-1 cells were pretreated with 50 ng/ml phorbol 12-myristate 13-acetate^41^ (PMA; Sigma-Aldrich) for 48 h^42^ for the macrophage differentiation. The PMA-treated THP-1 cells were then maintained in the PMA free growth medium for another 24 h.

### Cell viability test

Reagents of the LIVE/DEAD Cell Viability kit (cat# L-3224, Life Technologies) were added into the culture media for 20 min to stain with different fluorescence signals for live and dead cells (Fig. S1 in the supplementary material). Fluorescence images (TE300, Nikon, Tokyo, Japan) were then captured using an inverted fluorescence microscope (Zyla 4.2, Andor, Belfast, UK).

### Immune cell stimulation

Reagents, such as polyhydroxyalkanoates (PHA)^43^ and lipopolysaccharides (LPS),^44^ are widely adopted to stimulate the cytokine secretion of immune cells for their roles in the immune response. Here, we have chosen LPS (cat# L5886, Sigma-Aldrich) as the external stimulant. We applied LPS to the PMA- treated THP-1 cells in each test with a defined concentration of 0, 10, or 100 ng/ml. We then collected and quantified the cytokine concentration in the culture media of PMA-treated THP-1 cells at multiple time points (0, 2, 4, 6, 8, 10, and 12 h).

### Calibration by flow cytometry

The cytokine-sensitive fluorescence microbeads (cat# 551811, BD Biosciences) were calibrated using premixed human cytokines samples with known levels of IL-8, IL-1β, IL-6, IL-10, TNF, and IL-12p70. For each cytokine type, through diluting a standard solution (5000 pg/ml), multiple cytokine samples were prepared with different defined cytokine concentrations from 5 to 5000 pg/ml. Next, each of the diluted samples (volume: 50 *μ*l and concentrations: 5–5000 pg/ml) was incubated with antibody-conjugated detection microbeads (volume: 50 *μ*l and concentration: 10^3^ beads/*μ*l) and phycoerythrin-conjugated secondary antibodies (concentration: 10 *μ*g/ml) together for 3 h at room temperature to form a sandwich complex, of which the fluorescence intensity should reveal the cytokine concentration. After incubating and washing the microbeads with 1 ml phosphate-buffered saline (PBS), the spectrum of fluorescence intensity was investigated using flow cytometry (BD FACSVerse™, BD Biosciences, San Jose, CA, USA) with the allophycocyanin (APC) channel.

### Device fabrication

The device fabrication was based on replica molding of polydimethylisoxane (PDMS) as described in Fig. S2 in the supplementary material. Two silicon wafers were fabricated by photolithography with SU-8 photoresist (SU-8, Microchem) for microchannels in the upper control layer (SU-8 2010; height: 20 *μ*m) and the chamber layer at the bottom (SU-8 100; height: 100 *μ*m). For the middle flow layer (height: 20 *μ*m), another silicon wafer was patterned with AZ-50XT photoresist (AZ Electronic Materials) and then reflowed at 120 °C for 1 min after photolithography. All the mold surfaces was salinized with trichloro (1H, 1H, 2H, 2H-perfluoro-octyl)silane (Sigma-Aldrich) to facilitate the release of the molded PDMS layers in the later demolding step.

Polymethylsiloxane (PDMS; Sylgard-184, Dow Corning) was prepared by mixing the monomer and curing agent with a ratio of 10:1 for three layers of PDMS substrates. A PDMS substrate with a thickness of 5 mm was prepared by pouring the PDMS pre-polymer on the control layer mold. On the other hand, a layer of 35 *μ*m-thick PDMS was spincoated on the flow layer mold. After cutting and peeling off the control layer, its channel side and the chamber layer were treated with oxygen plasma (energy: 10 kJ; Harrick plasma cleaner PDC-002). These two layers were then aligned and bonded under a stereomicroscope. After cutting and peeling off the control-flow substrate, holes were then punched at the liquid inlets and outlets (diameter: 0.5 mm). Another PDMS substrate with a thickness of 1 mm was prepared by pouring the PDMS pre-polymer on the chamber layer mold. After baking and peeling off the chamber substrate, holes were punched on this layer for the detection chambers (diameter: 1 mm). The control-flow substrate was then bonded on the chamber substrate using the oxygen plasma. The entire stacked PDMS substrate was further bonded on a glass slide (width: 25 mm; length: 75 mm; and thickness: 170 *μ*m) using the oxygen plasma again. The microfluidic device was then baked at 75 °C for 2 h to ensure thorough crosslinking of PDMS.

### Device preparation

The microfluidic device was sterilized by baking at 100 °C for >10 h and applying UV exposure for >2 h. All the control channels were filled with distilled water. The syringe tubing (Tygon^TM^ tubing, US Plastics, Lima, OH) was connected to the control inlets of the device and the computer-controlled pressure supplies (pressure: 0–30 psi).^45,46^ Phycoerythrin-conjugated secondary antibody solution (∼50 *μ*g/ml), detection microbeads, and wash buffer was injected through the corresponding liquid inlets with the air pressure manifold (pressure: 0 or 0.2 psi). All the control valves were maintained with a pressure of 30 psi so that all the flow channels and detection chambers can be closed completely. The device was then placed on an inverted microscope (objectives: 20×; working distance: 0.35 mm; TE300, Nikon, Tokyo, Japan) installed with a camera (Zyla 4.2, Andor, Belfast, UK) and an environment-controlled chamber (37 °C and 5% CO_2_). The acquired fluorescence micrographs were a series of 1024 pixel × 1024 pixel 16-bit gray-scale images.

### Statistics

All error bars in plots are standard errors. *p*-values are determined by comparing two groups of data using Student's two-tailed, unpaired T-test. An asterisk in a plot represents a significant difference between two data groups (*p *<* *0.05).

## RESULTS AND DISCUSSION

### Influence of sample extraction volume

We investigated feasibility of functional microbead arrays for detection of cytokine concentrations in a cell culture with multiple time points by four experiment groups. In each of the experiments, we prepared the THP-1 cells (cell density: 1 × 10^6^ cells/ml) growing in 5 ml of media in a culture flask. Once the cells were further stimulated by LPS, we regularly extracted a volume of media from the cell culture to perform the cytokine quantification using the standard protocol at different time points (0, 2, 4, 6, 8, 10, and 12 h). It was expected that the detected cytokine levels should vary with time, as such dynamics of immune cells upon external stimuli can reflect some immune response characteristics. Afterwards, we examined the viability of the cells remaining in the flask. The first experiment group considered extracting a sample volume of 5 *μ*l in each cytokine measurement with refilling 5 *μ*l of fresh media; while the second group considered the same extraction volume but skipped the media refill. The sample will then be diluted by adding the fresh culture media with a volume of 45 *μ*l. The third and fourth groups were the same as the first two groups except that a larger extraction volume of 50 *μ*l was considered. An additional group was the control case included only the cell viability test but no medium extraction or cytokine detection steps. Furthermore, we have performed 5 repeated experiments for each of the above cases. There was no significant reduction in cell viability, defined as the percentage of live cells in the entire (live and dead) cell population, among all the five groups (Fig. S1 in the supplementary material).

**FIG. 1.**
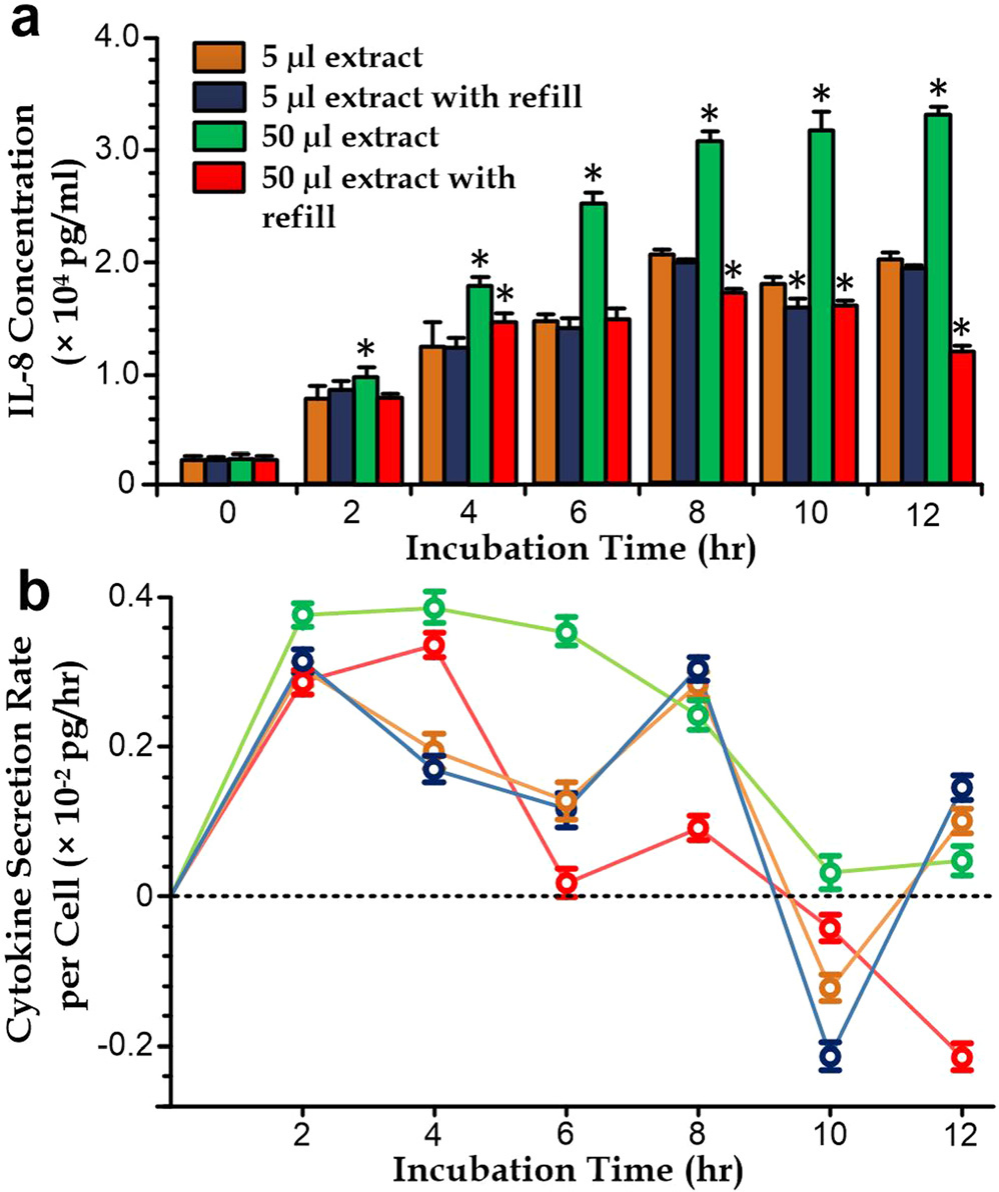
(a) IL-8 concentration in cell media after stimulation for 5 and 50 *μ*l extraction volumes in each measurement with and without media refill. The asterisk indicates a *p*-value < 0.05 compared to the case of 5 *μ*l without refill. (b) Cytokine secretion rate per cell (unit: pg/h).

The experiment groups correspond to different variations in the biochemical concentrations in the cell environment. The medium extraction step reduces the remaining medium volume with the immune cells; and therefore, the same cytokine secretion rate of the cells would then induce a relative higher cytokine concentration in the media. On the other hand, the media refill step would dilute the cytokine level in the media. In these experiments, we have further converted the detected cytokine concentrations at different time points [Fig. 1(a)] to the cytokine secretion rate (unit: pg/h per cell) among the first four experiment groups [Fig. 1(b)]. As the cytokine secretion profiles are significant different among the experiment cases, our results indicate that the different temporary cytokine levels can vary the cytokine secretion rates over the monitored experiment period. To eliminate the effects caused by the media extraction step, we need to consider largely reducing the sample volume in each cytokine detection. Yet, we have examined that the convention cytokine detection procedures using flow cytometry only supports an accuracy measurement for a minimal sample volume. Together, an alternative quantification method for the cytometric microbeads is required for the profiling of cell cytokine secretion dynamics.

### Microfluidic immunoassay operation

**FIG. 2.**
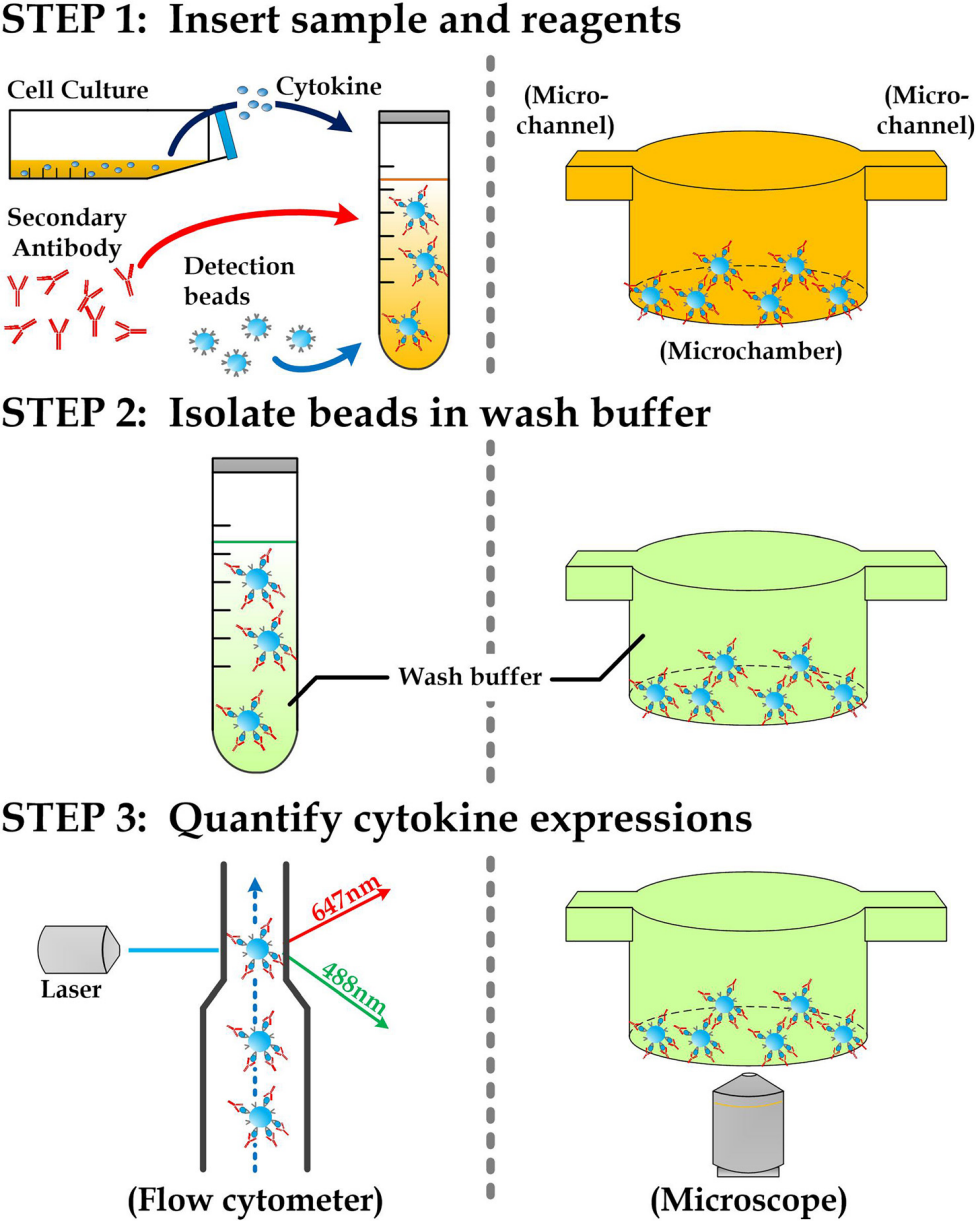
Detection schemes using flow cytometry (*left*) and proposed microfluidic procedures (*right*).

The goal of this work is to transform the standard protocol of a commercial cytometric microbead array (cat# 551811, BD Biosciences) into equivalent microfluidic procedures with a large reduction in required sample size and detection time. In typical microbead detections, as shown in Fig. 2 (*left*), microbeads coated with antibodies were pipetted and mixed up with bio-samples to capture target cytokine molecules on the bead surfaces, followed by the discharge of all the reagents and the addition of secondary antibody molecules modified with fluorescence. Accordingly, the multiple cytokine concentrations revealed by the captured cytokines on bead surfaces were then transferred to corresponding fluorescence intensities and quantified by various techniques such as flow cytometry. Similarly, we executed these procedures using microfluidics (Fig. 2, *right*), in which the required sample volume for each detection can be largely miniaturized from ∼50 *μ*l to <2 *μ*l. Briefly, we should fabricate an array of microchambers as the miniaturized syringe tubes in the conventional procedures for storage of extracted samples and other reagents. As the microbeads are confined in the microfluidic device, microfluidic mixers should be integrated with the microchambers for the required mixing process. For the same reason, we need to adopt another applicable imaging technique for quantifying fluorescence intensity of the microbeads in the microchambers.

**FIG. 3.**
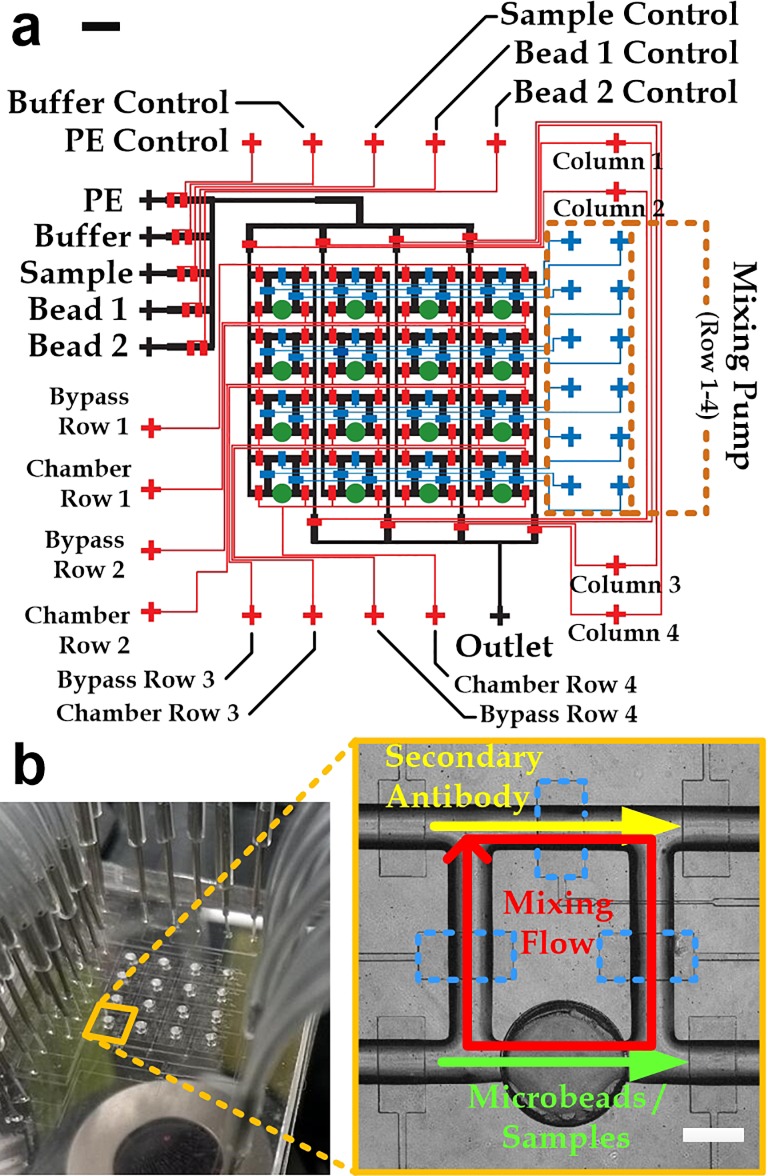
(a) Design of the microfluidic immunoassay device. Scale bar: 2 mm. (b) A fabricated device (*left*) and one detection region including a chamber and a mixing channel (*right*). There are three microvalves (*blue dotted boxes*) driving the mixing flow in the chamber region. Scale bar: 500 *μ*m.

Based on the above requirements, an integrated microfluidic device composed of an array of 4 × 4 detection regions was successfully designed [Fig. 3(a)] and fabricated [Fig. 3(b)]. This testing device is based on our recently reported work.^40^ Each detection region contains a microchamber (diameter: 1 mm; height: 1 mm; and volume: 0.79 *μ*l) and a bypass channel. The device contains three layers: control/mixing layer (*red/blue*), flow layer (*black*), and chamber layer (*green*). The control/mixing layer was above the flow layer; and the chamber layer was at the bottom. To measure the cytokine level, we first injected cytokine sensitive microbeads (diameter: 7 *μ*m) with a density of 10^3^ beads/*μ*l into a microchamber (diameter: 1 mm and depth: 1 mm) through multiple inlets controlled by different microvalves. Thus, there were ∼785 microbeads in each detection chamber. After 30 min, the microbeads could sink into the chamber bottom and be kept afterward. The microchamber and the microchannel in each detection region were then filled with the extracted samples and the phycoerythrin-conjugated fluorescence secondary antibodies, respectively.

After closing the inlet and outlet of pneumatic microvalves in the detection region, we incubated the microbeads with the biosample and reagents. Meanwhile, three microvalves [inset of Fig. 3(b)] along the microchannel of a mixer region were pressurized with a peristaltic sequence of the three pressurization patterns (on-on-off, off-on-on, and then on-off-on). The switching time between the pressurization patterns was set as 250 ms (Fig. S3 in the supplementary material). The microvalves also mixed the liquids in the mixing region based on the Taylor Dispersion effect.^47,48^ We then injected wash buffer (PBS) into the detection chamber and conducted the microbeads washing process for 30 s. Using the washed microbeads, corresponding fluorescence images for cytokine molecules (488 nm) and bead bodies (647 nm) were collected in order to measure the corresponding cytokine concentration and label the position of the bead body, respectively.

### Minimal incubation and mixing duration

**FIG. 4.**
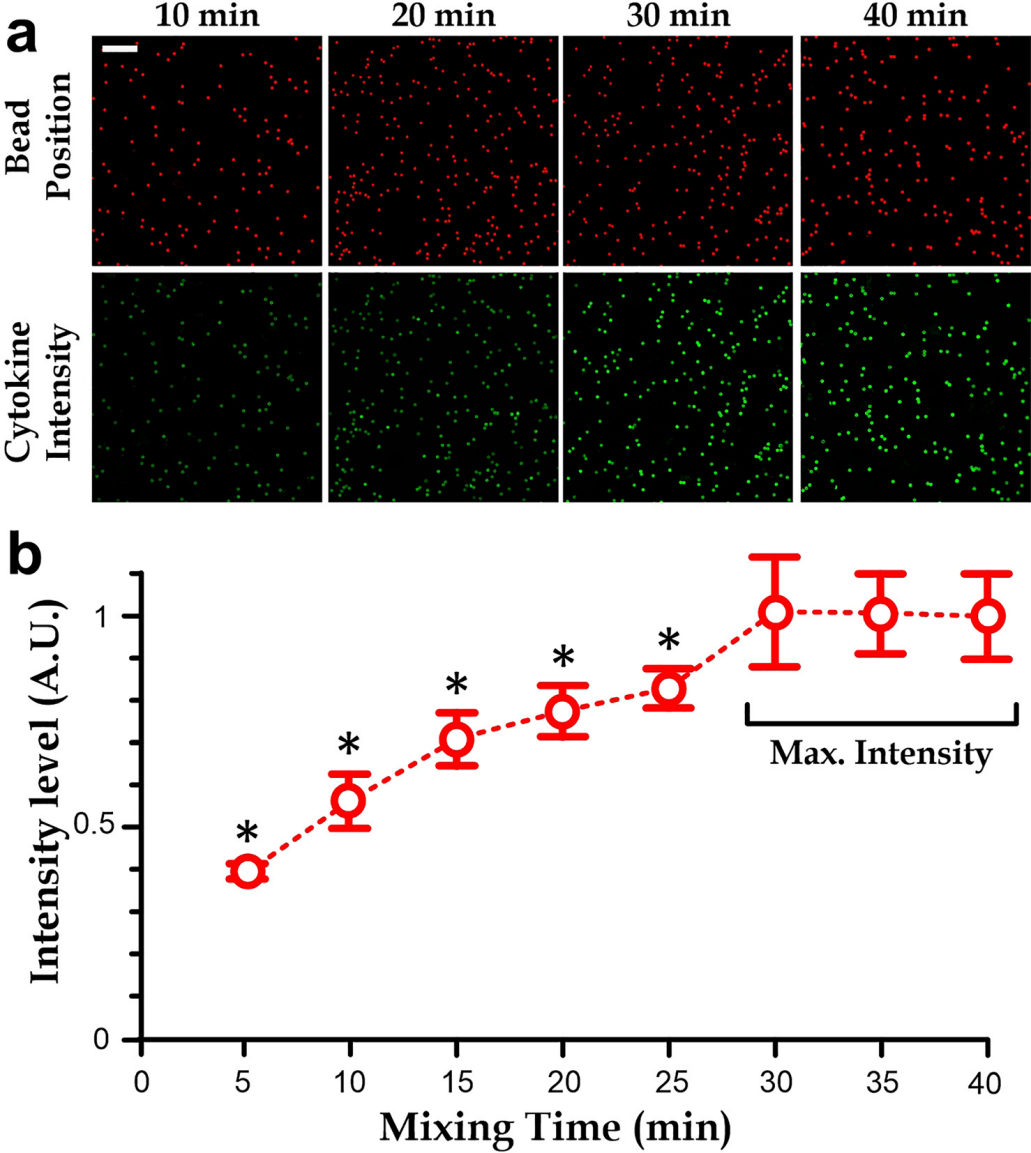
(a) Fluorescence images of IL-10 expressions on cytometric microbeads (*red*: bead body and *green*: stained cytokine intensity) at different mixing times. Scale bar: 100 *μ*m. (b) Stained cytokine intensity verses mixing time. Each data point is the average level of >8 repeated measurements. The asterisk indicates a *p*-value < 0.05 comparing to the intensity level of 40 min mixing time.

In the typical protocols of commercial cytometric microbeads, the procedures are rather tedious, including several times of washing and centrifugal steps; and the required incubation time is long (∼3 h) as the molecular transport is mainly governed by diffusion. The microfluidic operations of these microbeads can automate the required procedures. Further, the continuous microvalve-driven mixing can be applied during the incubation and therefore we expect a shorter incubation time. We are interested in investigating the required incubation duration of the microfluidic immunoassay for promising measurements. In the experiments, we have measured prepared cytokine solutions (IL-10) with a defined concentration (1250 pg/ml) for different incubation and mixing durations of the microfluidic immunoassay. We have considered eight mixing durations from 5 min to 40 min and the results. In each measurement, we captured the fluorescence images for the bead body (647 nm) and cytokine concentration (488 nm) [Fig. 4(a)]. We then only considered the average cytokine-related fluorescence intensity over the microbead region. The results (Fig. 4) indicate that an insufficient mixing time induces an underestimated cytokine values; and the mixing time of 30 min is sufficient for a promising measurement. In other words, the microfluidic implementation of the cytometric microbeads can shorten the measurement duration from 3 h to 30 min.

### Detection limit of cytometric microbeads in the microfluidic setting

**FIG. 5.**
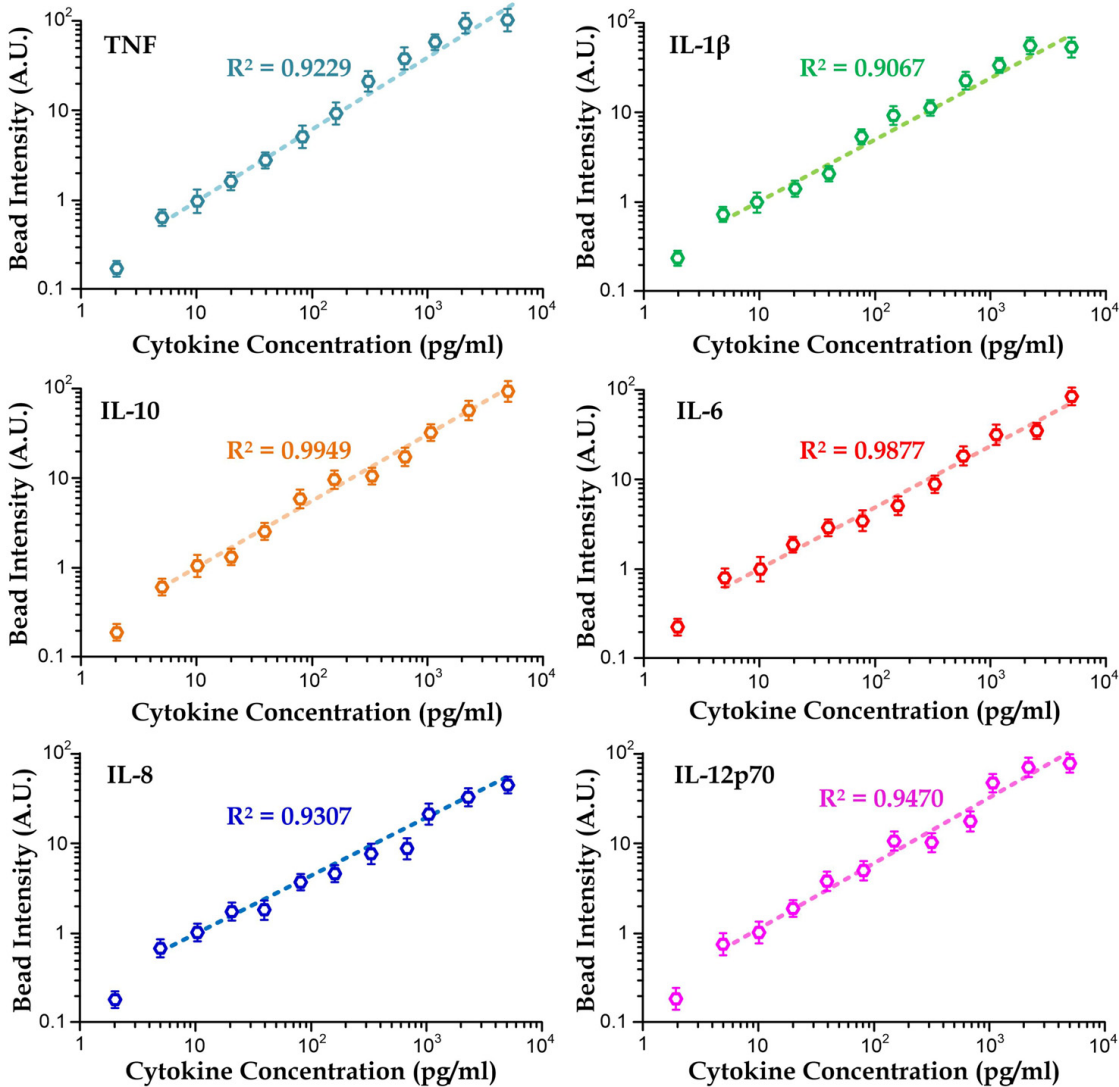
Calibration curves for six different cytometric microbeads in the microfluidic device. The data points for 2 pg/ml cytokine concentration are not included in the fitting line. Each data point was obtained from the average value of >5 repeated measurements.

We calibrated fluorescence intensity against cytokine concentration of six types of microbeads (TNF, IL-6, IL-8, IL-10, IL-1β, and IL-12p70) in order to determine the detection limit of measurement using the microfluidic immunoassay. We considered cytokine concentrations in the range of 1–5000 pg/ml. Our results (Fig. 5) exhibit the linearity (*R*^2^ > 0.9) of fluorescence intensity and cytokine concentration in the log scale, agreeing with the reported characteristics of cytometric microbeads. The cytokine detection limit in the microfluidic setting is ∼5 pg/ml, which is significantly better than the detection using flow cytometry (20 pg/ml). This improvement can be explained by the fact that the fluorescence intensity is obtained from all the captured microbeads in a fluorescence image rather than a single microbead as in the flow cytometry detection. Additionally, longer exposure duration (>200 ms) can be applied in the microfluidic measurement because microbeads are static in the microchambers, comparing to the shorter effective exposure time (∼60 ms) on the flowing microbeads during the flow cytometry operation.

### Profiling cytokine secretion dynamics of immune cells

**FIG. 6.**
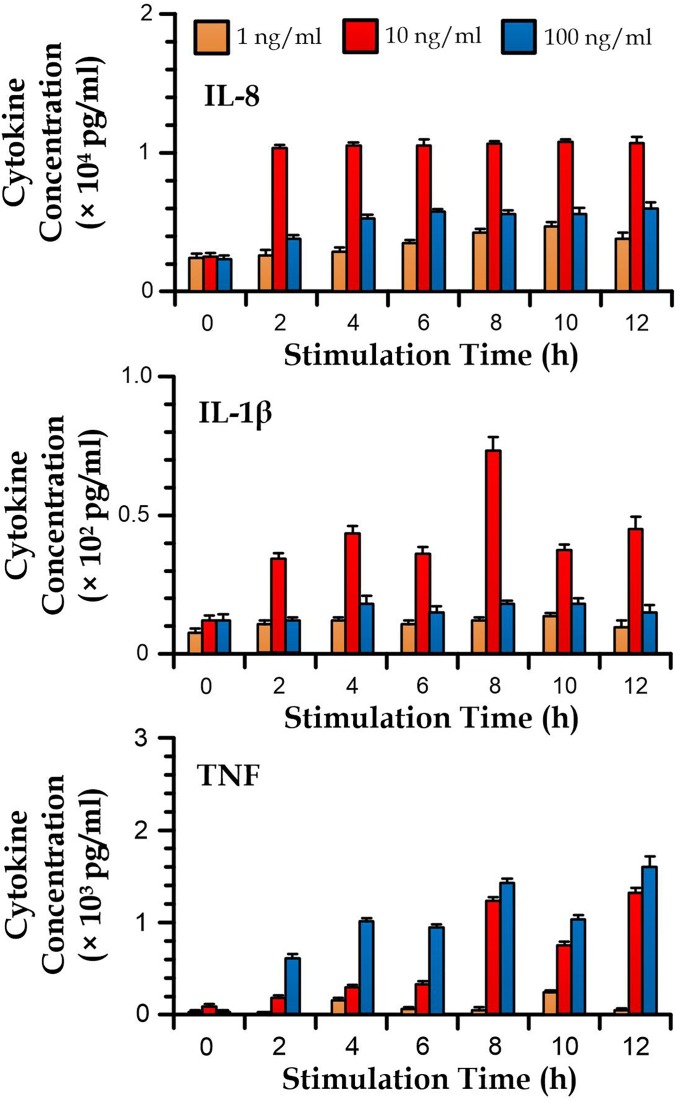
Concentrations of IL-8, IL-1β, and TNF in the media of PMA-treated THP-1 cells stimulated by different levels of LPS over 12 h. *N *>* *10 from four repeated cell stimulation experiments.

We have further applied the microfluidic immunoassay strategy to monitor the cytokine dynamics of the PMA-treated THP-1 cells stimulated by different levels of LPS (1, 10, and 100 ng/ml). We adopted the cells at the coverage of 1 × 10^6^ cells/ml growing in a 10 cm^2^ culture flask. The culture media was extracted from the flask with a volume of <5 *μ*l at each time point of 0, 2, 4, 6, 8, 10, and 12 h after stimulation. The media extracts were loaded into the microfluidic immunoassay for quantify three cytokines: IL-8, IL-1β, and TNF. Considering the required quantification involved 3 different cytokines at 7 time points, 21 detection chambers, and therefore 2 devices which were used in each measurement trial. Notably, as the IL-8 concentration can exceed the maximum detect range of the microbeads (5000 pg/ml) in some cases, those sample extracts were diluted 10 times before the measurement. The results (Fig. 6) reveal the effects of LPS on the secrete cytokines of the THP-1 cells. For instance, LPS can induce the secretion of IL-8, IL-1β, and TNF. The secretion of the three cytokines are, in general, with a higher rate for the cells stimulated by 10 ng/ml LPS, with statistically significant differences in the cytokine concentration between the 10 ng/ml LPS group and the other groups of the different LPS concentrations for all the cases in Fig. 6. It seems that the secretion of IL-8 and TNF can be stimulated with the highest effectiveness within a range of the stimulant concentration. It should be also highlighted that the microfluidic immunoassay offers promising monitoring of the dynamics IL-1β due to its lower detection limit (∼5 pg/ml) over the flow cytometry measurement (20 pg/ml).

**FIG. 7.**
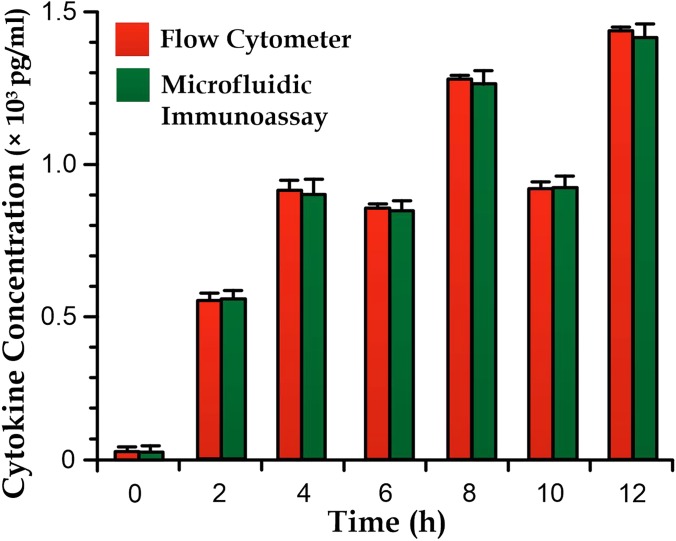
Measurement of microfluidic immunoassay (*N *>* *10) and flow cytometry (*N *≈* *1000) on TNF concentration in the cultured media of PMA-treated THP-1 cells stimulated by 10 ng/ml of LPS.

We have briefly confirmed the accuracy of cytokine concentrations measured by the microfluidic immunoassay. For the TNF monitoring, we extracted an extra sample volume of 5 *μ*l with a dilution to 50 *μ*l for the flow cytometry measurement. The measured values from both methods show a good agreement (no significant difference is observed), as shown in Fig. 7; and therefore, we believe that the microfluidic immunoassay strategy can offer an equivalent cytokine concentration value, but a lower detection limit than the flow cytometry.

## CONCLUSION

This research investigates the design of a microfluidic immunoassay device consisting of multiple detection regions for cytokine measurement using commercial functional cytometric microbeads. Such microfluidic strategy offers some key advantages over the microbeads quantification using flow cytometry. Despite the reduced reagent volume, the required biosample volume is largely reduced from 50 *μ*l to <5 *μ*l per measurement. We have examined that the reduction of the biosample extraction volume is important for reliable cytokine level monitoring. Assisted by the microfluidic mixing in the detection chamber, the processing time can be reduced from 3 h to ∼30 min. Through characterizing the microfluidic detection scheme with different design parameters, the cytokine detection limit of the microfluidic strategy can be improved (∼5 ng/ml), compared to flow cytometry (∼20 ng/ml). Additionally, we have exhibited the monitoring of cytokine secretion from PMA-treated THP-1 cells under the stimulation of LPS. Characterization on other design parameters (e.g., the concentration of microbeads injected in each microchamber) may help to further improve the microfluidic detection performance.

In essence, this work has demonstrated the procedures for obtaining the measurement parameters for the improved cytokine measurement using functional microbeads in the microfluidic device. Depending on the application requirements, this microfluidic strategy can be transferred to the measurement of other commercial available functional cytometric microbeads, and the microfluidic device can be configured with the required device dimensions and the number of detection regions. Therefore, we believe that this microfluidic strategy can be further applied in monitoring cell secretion of multiple cytokines as well as other molecules supported by any functional cytometric microbeads, for both biomedical applications and general cell research.

## SUPPLEMENTARY MATERIAL

See supplementary material for additional figures related to cell viability, device fabrication, microvalve switching frequency, and representative raw images for Figs. 5 and 6.
